# PD62 Judicialization Of Health In A Brazilian University Hospital: How Can We Reduce The Budget Impact?

**DOI:** 10.1017/S0266462324003209

**Published:** 2025-01-07

**Authors:** Mayra Carvalho Ribeiro, Giovana Fernanda Santos Fidelis, Claudio Lopes, Carlos Roberto Silveira Correa, Lucieni de Oliveira Conterno, Flávia de Oliveira Motta Maia, José Barreto Campello Carvalheira

## Abstract

**Introduction:**

Public hospitals in São Paulo can be held financially responsible for costs related to medications prescribed outside the recommendations of the Brazilian Unified Health System (SUS). The objective of this study was to describe these expenses in a public hospital and the measures implemented and evaluated to reduce this problem.

**Methods:**

In January 2023, the Health Technology Assessment Center collected data on legal proceedings filed against a tertiary teaching hospital from January 2021 to November 2023. The data were obtained from monthly reports sent by the São Paulo State Department of Health (SES). The proceedings were categorized according to the type of technology and its availability in the SUS, costs, and the prescribing specialties. The indicators developed were used to plan improvement actions to guide care teams, negotiate with the SES, and resolve current legal proceedings.

**Results:**

The cost of legal proceedings for 136 patients was BRL4,410,278 (USD890,965). Four medicines for six patients constituted 56 percent of the total cost. A group created an informative folder explaining how to access the National List of Essential Medicines, prescribe medicines from the high-cost program, and make administrative requests. Other related actions were the creation of a standardized process for requesting medicines, monthly assessment of judicialization data, clinical discussions with prescribers, and educational activities with residents. The interventions reduced average monthly costs from BRL177,268 (USD35,811) to BRL85,493 (USD17,271) in the last trimester.

**Conclusions:**

Knowledge and measurement of judicialization costs allowed the hospital to implement improvements to help avoid new legal proceedings and to understand the demands of medical specialties regarding situations not covered by SUS guidelines. The Health Technology Assessment Center’s work with managers made it possible to identify opportunities for improving the education of professionals regarding the procedures and technologies available in the SUS.

